# Supplementary material for: Chronic hepatitis B in remote, tropical Australia; successes and challenges
Source: PLoS One. 2020 Sep 3;15(9):e0238719. doi: 10.1371/journal.pone.0238719 (PMC7470305; doi:10.1371/journal.pone.0238719)
Supplement: S1 Table — (DOCX) [file pone.0238719.s001.docx]

**S1 Table:** Country of birth of individuals living in rural FNQ living with CHB

| **Country of birth** | **Number (%)** |
| --- | --- |
| Australia | 545 (91%) |
| Papua New Guinea | 27 (4%) |
| Philippines | 3 (0.5%) |
| Cook Islands | 2 (0.3%) |
| Fiji | 2 (0.3%) |
| Germany | 2 (0.3%) |
| Indonesia | 2 (0.3%) |
| Macau | 1 (0.2%) |
| New Zealand | 1 (0.2%) |
| Switzerland | 1 (0.2%) |
| Tonga | 1 (0.2%) |
| Outside Australia | 12 (2%) |
| Not stated | 3 (0.5%) |
