# Supplementary material for: Chronic hepatitis B in remote, tropical Australia; successes and challenges
Source: PLoS One. 2020 Sep 3;15(9):e0238719. doi: 10.1371/journal.pone.0238719 (PMC7470305; doi:10.1371/journal.pone.0238719)
Supplement: S1 File — (PDF) [file pone.0238719.s002.pdf]

Managing chronic hepatitis B in pregnancy

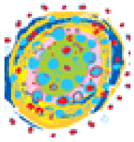

Please refer all pregnant women with chronic hepatitis B (HBsAG positive) to a Medical Officer with Hepatitis B s100 prescribing rights or **Cairns and Hinterland HHS Liver Clinic ph 42266669 or 42267224.**

The following hepatitis B tests need to be completed at the first available opportunity. (first and second trimester) to determine management.
HBsAg / HBeAg / HBeAb, HBV DNA, FBC / Chem 20 (LFT, Creatinine) / INR

↓

Refer to Medical Officer Hepatitis B s100 prescriber or Cairns and Hinterland HHS Liver Clinic, phone 42266669 or 42267224.

Management of baby born to mother with chronic hepatitis B

At delivery
Hepatitis B Immunoglobulin and
Hepatitis B vaccine within 12 hours of delivery
Breastfeeding recommended

↓

Routine Hepatitis B vaccination as per Immunisation Schedule QLD.

↓

Check baby HBsAg, HbcAb at 9-12 months. (0.6mL SST tube)

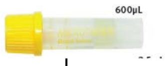

↓

Results = HBsAG positive

Refer to Paediatrician

↓

Results =
HBsAg negative
HBsAB > 10 international units/ml
Hep B Immune
No further Action

↓

HBsAg Negative
HbsAb <10 international units / ml
Give Hepatitis B vaccine booster and retest HBsAb after 4-6 weeks

→

HbsAb > 10 international units / ml
Hep B Immune
No further Action

HbsAb <10 international units / ml
Discuss with Cairns Hospital Liver clinic or Paediatrician

Reference
https://ashm.org.au/resources/HBV-Resources-list/hepatitis-b-and-primary-care-providers/
